# Low fat diet versus low carbohydrate diet for management of non-alcohol fatty liver disease: A systematic review

**DOI:** 10.3389/fnut.2022.987921

**Published:** 2022-08-16

**Authors:** Hamed Kord Varkaneh, Faezeh Poursoleiman, Mohammad Khaldoun Al Masri, Kamar Allayl Alras, Yamen Shayah, Mohd Diya Masmoum, Fulwah Abdulaziz Alangari, Abd Alfatah Alras, Giulia Rinaldi, Andrew S. Day, Azita Hekmatdoost, Ahmed Abu-Zaid, Emad Kutbi

**Affiliations:** ^1^Student Research Committee, Department of Clinical Nutrition and Dietetics, Faculty of Nutrition and Food Technology, Shahid Beheshti University of Medical Sciences, Tehran, Iran; ^2^College of Medicine, Alfaisal University, Riyadh, Saudi Arabia; ^3^College of Kinesiology, University of Saskatchewan, Saskatoon, SK, Canada; ^4^St. George's Hospital, London, United Kingdom; ^5^Department of Paediatrics, University of Otago, Dunedin, New Zealand; ^6^Department Pharmacology, College of Graduate Health Sciences, The University of Tennessee Health Science Center, Memphis, TN, United States; ^7^Department of Biorepository, Biomedical Research Administration, King Fahad Medical City, Riyadh, Saudi Arabia

**Keywords:** non-alcoholic fatty liver disease, low fat diet, low carbohydrate diet, NAFLD, liver fat

## Abstract

Although there is a consensus on beneficial effects of a low calorie diet in management of non-alcoholic fatty liver disease, the optimal composition of diet has not yet been elucidated. The aim of this review is to summarize the results of current randomized controlled trials evaluating the effects of low fat diet (LFD) vs. low carbohydrate diet (LCD) on NAFLD. This is a systematic review of all the available data reported in published clinical trials up to February 2022. The methodological quality of eligible studies was assessed, and data were presented aiming specific standard measurements. A total of 15 clinical trial studies were included in this systematic review. There is an overall lack of consensus on which dietary intervention is the most beneficial for NAFLD patients. There is also an overall lack of consensus on the definition of the different restrictive diets and the percentage of macronutrient restriction recommended. It seems that low calorie diets, regardless of their fat and carbohydrate composition, are efficient for liver enzyme reduction. Both LCD and LFD have similar effects on liver enzymes change; however, this improvement tends to be more marked in LFD. All calorie restrictive dietary interventions are beneficial for reducing weight, liver fat content and liver enzymes in individuals with NAFLD. Low fat diets seem to be markedly successful in reducing transaminase levels. Further research is needed to explore diet intensity, duration and long-term outcome.

## Introduction

Non-alcoholic fatty liver disease (NAFLD) is one of the most prevalent liver diseases worldwide (20–30%) (1). Individuals with NAFLD may develop liver injury with a subset developing progressive fibrosis (non-alcoholic steatohepatitis), cirrhosis and complications including end-stage liver failure and hepatocellular carcinoma (2). Fatty liver is one of the causes of liver transplantation (3). In many aspects, the pathophysiology of NAFLD is similar to that of obesity, dyslipidemia and diabetes (4).

Diet and exercise are first line treatments for NAFLD. Studies have shown that patients with NAFLD consume excessive amounts of total energy, refined carbohydrates (including fructose), fibers and antioxidants (vitamin C and vitamin E), cholesterol and saturated fats (SFA) with an insufficient intake of polyunsaturated fats (PUFA) (5, 6). Since there is no pharmacotherapy currently available for patients with NAFLD, lifestyle changes remain the fundamental management option (7, 8). In patients with NAFLD (overweight and obese), calorie restriction drives the reduction of liver fat, body weight, and histological improvement of non-alcoholic steatohepatitis (NASH) (9, 10). However, there is conflicting data on which hypocaloric dietary plan should be adopted according to the macronutrient composition (11, 12). Nordmann et al. (13) showed that low-fat diets are more effective than low-carbohydrate diets in reducing total cholesterol (TC) and low-density lipoprotein (LDL) cholesterol concentrations. In contrast, low-carbohydrate diets can be more effective than low-fat diets in increasing high-density lipoprotein (HDL) cholesterol concentrations and reducing triglyceride (TG) and transaminase levels with a further decrease in 24-h circulating blood insulin concentrations in both isocaloric and hypocaloric conditions (14–17).

Current evidence suggests a change in diet composition alone can reduce hepatic fat infiltration. Studies in animal models and humans have shown that reducing intake of carbohydrate (CHO) sources such as added sugars, high glycemic grains, and fructose may be an effective approach to reverse fatty liver by significantly reducing insulin resistance and inflammation (18–20). A randomized clinical trial in adults demonstrated that a CHO restricted diet (<20 g/day) compared to a low-fat diet resulted in similar weight loss but greater reduction in hepatic fat (−55 vs. −28%, *p* < 0.001) (21). This suggests a clear metabolic advantage for CHO-restriction, independent of overall weight loss, in adults with NAFLD. A study showed that a low-carbohydrate diet was more effective than a low-fat diet in improving obesity (22, 23). Moreover, another paper explored the impacts of low carbohydrate vs. low-fat/low-calorie diets in achieving weight control and found that the low-carbohydrate diet is more effective than the low-fat/low-calorie diet (24). Furthermore, low-fat diets effectively reduce intrahepatic fat content (24). NAFLD is a significant independent risk factor for cardiovascular disease and type 2 diabetes because of concurrent dyslipidemia and insulin resistance (25), therapeutic diets are believed to be effective for reversal of changes seen with NAFLD (26).

However, despite these data, there is little evidence surrounding an all-inclusive diet for NAFLD. This systematic review focuses on randomized controlled trials (RCTs) of low carbohydrate diets compared to low fat diets, to assess their impact on NAFLD and liver enzymes.

## Methods

### Conduct of systematic review

A meta-analysis was conducted based on the Preferred Reporting Items for Systematic reviews and Meta-Analyses (PRISMA) statement.

### Search strategy

PubMed/Medline, Web of science, Scopus, and Cochrane library databases were searched for relevant articles about the intervention low fat diet or low carbohydrate diet in patients with NAFLD published up to February 2022; without any language restriction. The combination of MESH and non-MESH keywords were used (Supplementary Table 1). Furthermore, all reference lists of related articles, reviews, and meta-analyses were manually checked to avoid missing any studies. The quality of eligible trials was evaluated using the Cochrane quality assessment tool (27), which is comprised of the following: allocation concealment, blinding of participants and personnel, random sequence generation, incomplete outcome data, blinding of outcome assessment, selective reporting and other probable sources of biases (Table 1).

**Table 1:** The risk of bias in studies included in this study.

	**Random Sequence Generation (selection bias)**	**Allocation concealment (selection bias)**	**Blinding of participants and personnel (performance bias)**	**Blinding of outcome assessment (detection bias)**	**Incomplete outcome data (attrition bias)**	**Selective reporting (reporting bias)**	**Other bias**	**AHRQ standards**
Rodríguez-Hernández et al. (28)	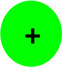	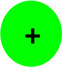	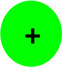	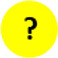	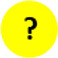	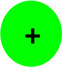	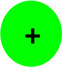	Fair
Arefhosseini et al. (29)	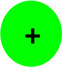	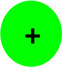	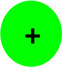	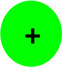	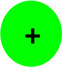	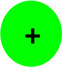	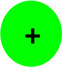	Good
Haufe et al. (23)	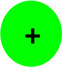	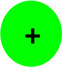	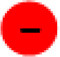	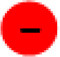	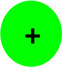	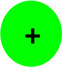	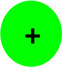	Poor
De Luis et al. (30)	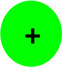	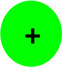	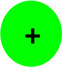	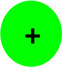	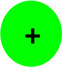	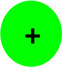	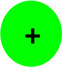	Good
Kirk et al. (14)	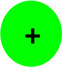	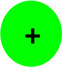	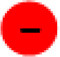	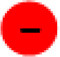	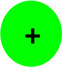	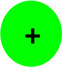	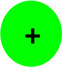	Poor
Marina et al. (31)	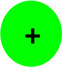	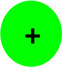	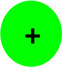	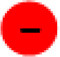	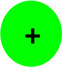	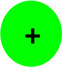	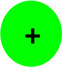	Poor
Kani et al. (26)	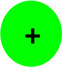	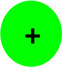	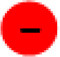	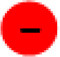	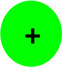	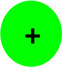	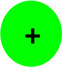	Poor
Browning et al. (21)	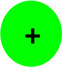	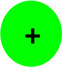	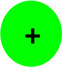	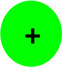	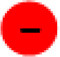	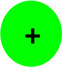	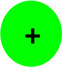	Poor
Benjaminov et al. (32)	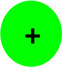	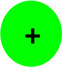	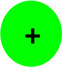	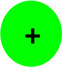	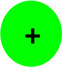	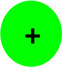	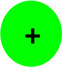	Good
Mardinoglu et al. (25)	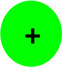	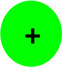	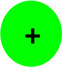	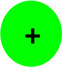	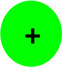	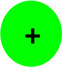	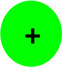	Good
Yu et al. (33)	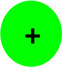	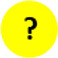	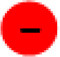	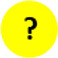	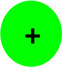	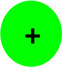	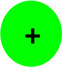	Poor
Biolato et al. (34)	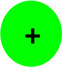	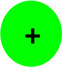	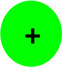	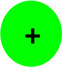	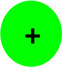	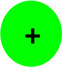	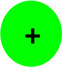	Good
Properzi et al. (35)	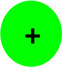	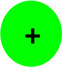	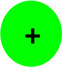	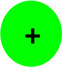	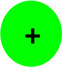	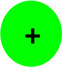	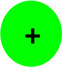	Good
Ryan et al. (36)	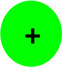	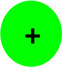	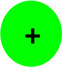	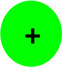	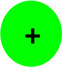	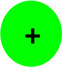	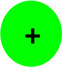	Fair
Ravansha et al. (37)	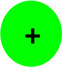	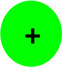	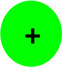	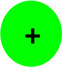	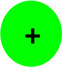	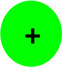	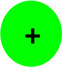	Good

### Eligibility criteria

The following criteria were regarded to select eligible studies based on PICO; (1) participants (p): studies that included adult subjects (≥18 years of age) with NAFLD, (2) Intervention (I): examined the effects of low fat diet or low carbohydrate diet in NAFLD patients, (3) comparison: compared with control or baseline value, (4) outcome: those that reported sufficient data related to liver. In addition, studies were excluded if they; (1) were executed on children, pregnant women, other diseases or animals, (2) were not trials, (3) did not provide sufficient information of NAFLD outcomes, or (4) investigated the effects of low fat diet or low carbohydrate diet along with other dietary changes. Unpublished documents and gray literature like conference papers, pilot studies, dissertations, and patents were also excluded.

### Data extraction

Two independent investigators (FP and HKV) undertook the study selection whereas a chief investigator (AH) provided further comment if any disagreements. Contact was made with the corresponding authors of any studies where insufficient data was available.

The following data was extracted from eligible studies: first author's name, year of publication and journal, study country, gender of participants, mean age, number of subjects in each group, trial duration, type and energy content of each diet intervention with percent of macronutrient, study design, and the mean and standard deviation of outcome measures at baseline and the end-of-trial.

#### Definition of low carbohydrate and low fat diet

Low carbohydrate diet definition is very inconsistent, but according to Seidelmann et al. new study (38) optimal proportion of carbohydrate for a healthy diet is 50–55%. Therefore, we considered ≤ 50% as a low carbohydrate diet (LCD) and according to other studies (13, 39) we defined low fat diet (LFD) as a maximum of 30% of the calorie intake from fat.

## Results

### Literature search and study characteristics

**Figure 1:**
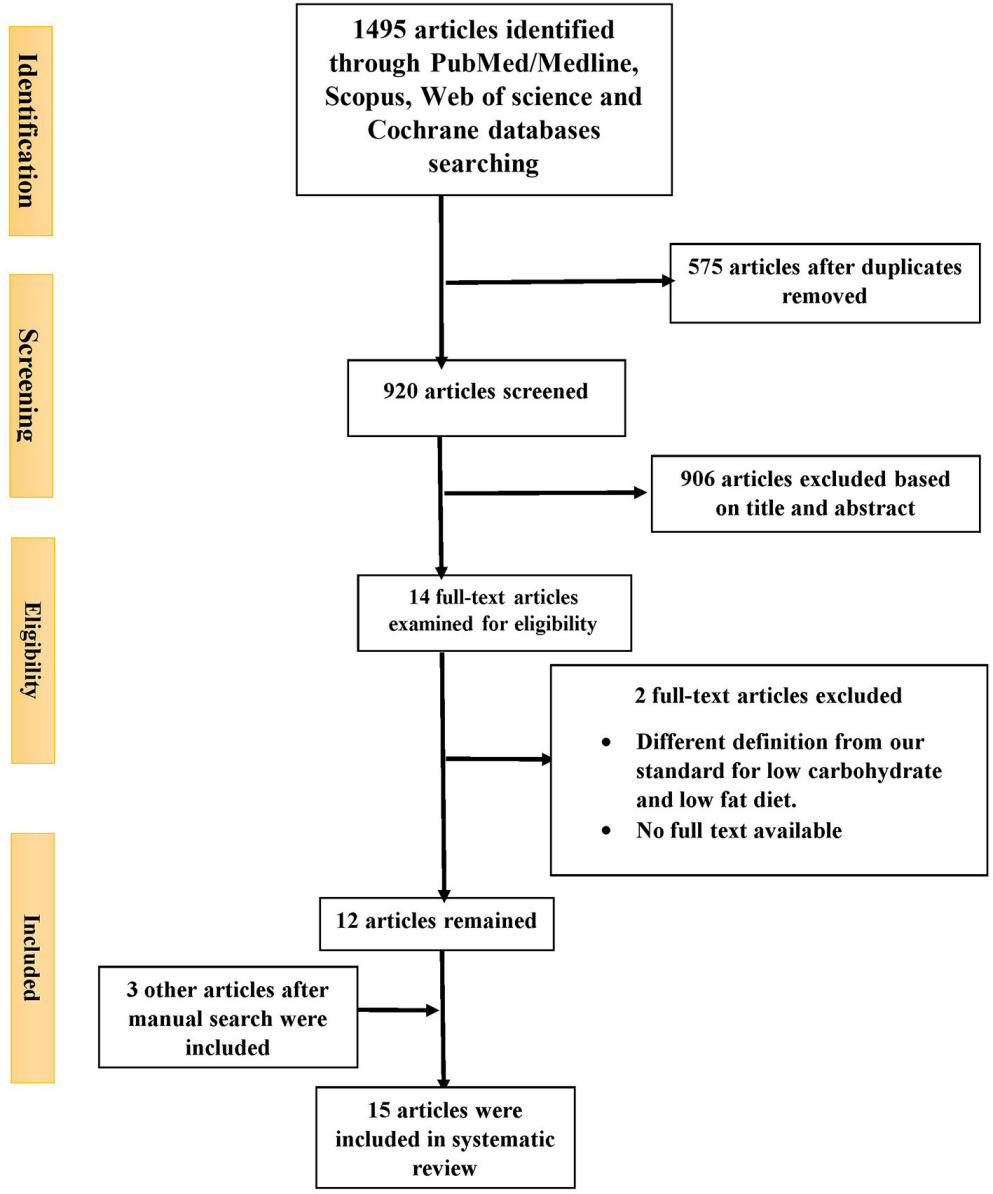
PRISMA flowchart for study examined and included into the meta-analysis.

Out of 1,495 articles identified from PubMed/Medline (*n* = 375), Scopus (474), Web of Science (465) and Cochrane databases (*n* = 181), 575 duplicate articles were excluded. A further 906 were excluded based on the title and abstract screening approach. The remaining 14 articles were reviewed with two independent authors by reading the full text. Two additional studied were excluded for the following reasons: no full text available (40) and different definition for low carbohydrate and low fat diet (low fat diet; ≤ 30% of total calorie from fat and low carbohydrate diet; ≤ 50% of total calorie from carbohydrate) (4) (Figure 1). After manual search three other articles were also included (14, 29, 32) (Table 2). Finally, 15 articles were included in the current study. Five studies reported LCD vs. other dietary patterns (21, 25, 26, 33), four reported LFD vs. other dietary patterns (34–37), six reported LCD vs. LFD (14, 23, 28–31) and from them nine reported the effects of LCD or LFD on liver fat content (14, 21, 23, 25, 31–33, 35, 36). Fifteen included studies were published between 2005 and 2019. Sample sizes varied from 8 to 140 patients across studies. Mean ages and mean baseline BMIs ranged from 32 to 55 years and 28–45.9 kg.m^2^, respectively. One trial was performed exclusively in women (28) and others included both genders. Intervention periods were as wide as 2–24 weeks. According to dietary interventions, carbohydrate percentage in LCD was varied from 8 to 45% and 15 to 30% for fat content in LFD.

**Table 2:** The characteristics of the 15 eligible studies.

**References, Country**	**Subjects**	**Diagnosis method**	**Study design**	**Follow up (weeks)**	**intervention**	**Control**	**Results**
**Low fat vs. low carb diet**							
Rodríguez-Hernández et al. (28), Mexico	Obese & NAFLD	Ultrasonography	Randomized non-controlled crossover trial	24	Low-fat diet (Fat: 21%, Carb: 54%)	Low-carb diet (Carb: 45%, Fat: 28%)	ALT and AST decreased more in the Low-carb diet but not significantly
Arefhosseini et al. (29), Iran	Overweight & NAFLD	Ultrasonography	Randomized controlled crossover trial	6	Low calorie low fat diet (Fat: 25%, Carb: 55%, minus 500 kcal)	Low calorie low carb diet (Carb: 40%, Fat: 40%, minus 500 kcal)	AST decreased in both group but more in LFD weight decreased significantly in both groups
Haufe et al. (23), Germany	Obese & high IHL	MRS	Randomized controlled crossover trial	24	Low calorie low fat diet (Fat ≤ 20%, minus 30% of energy intake before diet)	Low calorie low carb diet (Carb ≤ 90 g/day, Fat ≥ 30%, minus 30% of energy intake before diet)	Both hypocaloric diets decreased liver enzymes, body weight, and liver fat content similarly
De Luis et al. (30)	NAFLD	BMI≥30 ALT≥43	Randomized controlled trial	12	Low calorie low fat diet (Fat: 27%, Carb: 53%, 1,500 kcal)	Low calorie low carb diet (Carb: 38%, Fat: 36%, 1,507 kcal)	ALT and AST decreased more in the low-fat diet weight loss in groups were the same
Kirk et al. (14), USA	Obese	MRS	Randomized controlled trial	11	Low calorie low carb diet (Carb ≤ 50 g/day ~10%, Fat: 75%, minus 1,000 kcal)	Low calorie high carb diet (Fat: 20%, Carb ≥180 g/day ~ 65%, minus 1,000 kcal)	• ALT and AST did not changed. • Liver fat content decreases in low carb at 48 h but not in 11 weeks.
Marina et al. (31), USA	Overweight/obese	BMI > 27MRS	Randomized controlled trial	4	Low fat diet (fat: 20%, carb: 62%)	High fat diet (carb: 27%, fat: 55%)	• ALT, AST and Body weight had no changes in groups. • Liver fat decreased in LFD but not significantly.
**Low carb diet**							
Kani et al. (26), Iran	NAFLD	Ultrasonography	Randomized parallel trial	8	Low calorie low carb diet (carb: 45%, fat: 35%, minus 200–500 kcal)	Low calorie diet (carb: 55%, fat: 30%, minus 200–500 kcal)	ALT and ALT reduction and weight loss were the same in both group
Browning et al. (21), Texas	NAFLD	Liver Biopsy	Randomized crossover trial	2	Low carb diet (Carb> 25 kcal/kg of ideal body weight, ~8%, fat~ 59%)	Low calorie diet (fat~34%, car~50%, 1,200 kcal for women and 1,500 kcal for men)	• ALT reduction was not significant in either group but AST reduced significantly in both. • Weight were similar loss in both group. • Liver fat content reduction in LCD group was more
Benjaminov et al. (32), Israel	Obese	CT	Single arm trial	4	Low carb diet (carb: 14%, fat: 56%	–	• ALT and AST did not changed. Weight loss was significant • Reduction of liver fat content and size.
Mardinoglu et al. (25), Sweden	Obese & NAFLD	Biopsy	Single arm trial	2	Low-carb diet (carb <10%)	–	Liver fat content was dramatically reduced
Yu et al. (33), China	Obese & NAFLD	BMI > 30 liver fat >5.6%	Single arm trial	8	Low calorie low carb diet (carb <10%, minus 800 kcal)	–	Caloric restriction reduced liver fat content and body weight
**Low fat diet**							
Biolato et al. (34), Itlay	NAFLD	Biopsy-verified NAFLD and increased transaminases	Open-label Crossover trial	16	Low calorie low fat diet (fat: 18%, carb: 62%, 1,400 kcal)	Low calorie Mediterranean diet (carb: 40%, fat: 40%, 1,400 kcal)	Significant weight loss and ALT and AST reduction observed after MD
Properzi et al. (35), Australia	NAFLD	MRS	Randomized parallel trial	12	Low fat diet (fat: 30%, carb: 50%)	Mediterranean diet (carb: 40%, fat: 40%)	• ALT and GGT decreased significantly in both groups as well as similar weight loss. • No changes were observed in liver fat.
Ryan et al. (36), Australia	NAFLD	Biopsy	Randomized crossover trial	6	Mediterranean diet (carb: 40%, fat: 40%)	Low fat diet (fat: 30%, carb: 50%)	• Transaminases (ALT, GGT) level and weight loss changes were not significant and also not different between groups. • A significantly greater decrease in liver fat content was seen in MD group.
Ravansha et al. (37), Iran	Obese & NAFLD	BMI > 25 Ultrasonography	Single arm trial	6	Low calorie low fat diet (fat 25–30%, carb: 55–60%, minus 500-1,000 kcal)	–	Compared to the baseline decreased body weight and mean serum ALP, ALT, AST level were seen

NAFLD, non-alcoholic fatty liver disease; IHL, Intrahepatic Lipid; ALT, alanine aminotransferase; AST, aspartate aminotransferase; ALP, Alkaline phosphatase; GGT, Gamma-glutamyl transferase; carb, carbohydrate; MRS, magnetic resonance spectroscopy; carb, carbohydrate; MD, Mediterranean diet; LFD, low fat diet; LCD, low carbohydrate diet.

#### Quality assessment

The results of the quality assessment of the eligible studies are presented in Table 1. Most studies had a good quality, two had fair quality (28, 36), and six had a poor quality (14, 21, 23, 26, 31, 33) (an unclear risk of bias for in the allocation concealment and blinding of outcome assessments).

##### Effects of LCD or LFD on liver enzymes and body weight

###### LCD vs. other dietary patterns.

Two studies compared LCD (<30% from total calorie intake) with low calorie diet. Kani et al. (26) conducted a randomized parallel trial for 30 patients with NAFLD. After intervention with low calorie (−200 to 500 kcal) and low calorie LCD (−200 to 500 kcal, CHO: 45%, FAT: 35%) diets, both ALT and AST decreased. ALP reduction was only seen in low calorie group. However, the changes in liver enzymes and body weight were not significantly different between these two groups.

Browning et al. (21) compared a very LCD (CHO: 8%, FAT: 59%) with low calorie diet (CHO: 50%, FAT: 34%) in 18 patients with NAFLD. A similar weight loss was observed in both groups. Regardless of the dietary intervention, AST but not ALT changed significantly after 2 weeks weight loss.

In a single arm study, Benjaminov et al. (32) showed that a 4 week intervention with a very LCD (CHO: 14%, FAT: 56%) did not alter ALT and AST. Significant weight reduction was seen. Yu et al. (33) conducted a low calorie, very LFD (CHO <10%, 800 kcal) intervention for patients with obesity and NAFLD. In this single arm study, body mean weight reduction after 8 weeks was 6.8 kg (7% of the pre-intervention weights) (*p* = 0.001).

##### LFD vs. other dietary patterns

From four studies, three of them compared LFD with Mediterranean diet and one study had no control group. In a crossover study, 20 patients with NAFLD underwent 16 weeks of MD (CHO: 40%, FAT: 40%), 16 weeks of wash-out period, and 16 weeks of LFD (FAT: 18%, CHO: 62%) (34). Both diet interventions were calorie restricted (1,400 kcal). At the end of the MD period, significant weight loss and ALT and AST reduction were observed. But in the LFD group, there were no significant changes in body weight or transaminases. Serum transaminases and body weight at the beginning of LFD period were the same as the end point of MD period: consequently the conditions at the start of the LFD period differed from that of the MD group. These results may reflect the sequence of the dietary intervention.

Properzi et al. (35) compared LFD (FAT: 30%, CHO: 50%) and MD (CHO: 40%, FAT: 35–40%) in a parallel-group RCT. Fifty-six patients with NAFLD were recruited and after 12 weeks intervention data from 49 subjects were available for analysis. At the end of the study, ALT and GGT decreased significantly in both groups. Similar weight loss amounts were seen also, with no no significant differences seen at the final analysis. In a study by Ryan et al. (36) patients with NAFLD randomly received MD (CHO: 40%, FAT: 40%) or LF-HCD (FAT: 30%, CHO: 50%) for 6 weeks. After a 6 week washout period, the subjects swapped to the second diet. ALT and GGT changes were not appreciated in either diet. Similarly, weight loss was not different between two groups (*p* = 0.22).

Ravanshad et al. (37) designed a single arm randomized trial for obese patients with NAFLD.A low calorie LFD (FAT: 25–30%, CHO: 55–60%) for 6 weeks resulted in decreased body weight and mean serum ALP, ALT, AST levels. Although this study appeared to show beneficial effects for LFD with restricted calorie, the differential effects of calorie and fat restriction were able to be demonstrated.

###### LCD vs. LFD

Six interventional studies evaluated liver enzymes levels by comparing LCD and LFD. Rodríguez-Hernández et al. (28) enrolled 31 obese women with NAFLD in a randomized crossover study. They showed that 24 weeks of LCD (CHO: 45%, FAT: 28%) decreased ALT and AST more than LFD (FAT: 21%, CHO: 54%), but there was no statistical significance. Since both diets were hypocaloric, the authors claimed that weight loss diminished aminotransferase levels regardless of fat or carbohydrate percentage.

Arefhosseini et al. (29) compared LCD (CHO: 40%, FAT: 40%) with LFD (FAT: 25%, CHO: 55%) in 44 overweight patients with NAFLD patients over 6 weeks. The results arising showed that, regardless of the type of diet, calorie deficit (−500 kcal/day) can reduce AST. Unlike the study conducted by Hernández et al. (28), it was only significant for the LFD. Haufe et al. (23) randomized 102 obese patients including 45% with NAFLD into a LCD (CHO: 25%, FAT 45%) and a LFD (FAT: 27%, CHO: 52%). Both diets were hypocaloric and weight loss was observed in both groups. There were no significant differences in liver enzyme reduction between groups. De Luis et al. (30) assessed 12 weeks of hypocaloric diet with LFD (FAT: 27%, CHO: 53%) and LCD (CHO: 38%, FAT: 36%). Diet therapy improved ALT and GGT inboth groups, but AST decreased only with LFD. Compared with LCD, AST and ALT levels were significantly reduced in the LFD group. Weight losses were the same in both groups (−4 kg).

Kirk et al. (14) designed a randomized clinical trial to compare two types of diet: high carbohydrate diet (=LFD) (FAT: 20%, CHO: 65%) and LCD (CHO: 10%, FAT: 75%). Twenty-two obese patients were enrolled and about 50% of them also had NAFLD. Both diets were hypocaloric (−1,000 kcal/day). After 48 h and ~11 weeks dieting, ALT and AST did not change with either diet. Regardless of the diet group, all subjects lost weight. In a similar study design, Marina et al. (31) evaluated a high fat diet (=LCD) (CHO: 27%, FAT: 55%) and LFD (FAT: 20%, CHO: 62%). About 54% of patients were NAFLD. No changes in liver enzymes or body weight were seen between groups but there was an increasing trend for GGT in LFD.

Overall, high heterogeneity in carbohydrate and fat percent was observed in the designed diets. Low carbohydrate and low fat diets are not well-defined and there is no consensus definition for them and as mentioned in studies percent from total calorie is ranged 8–45% for low carbohydrate diet and 15–30% for low fat diet. Furthermore, most studies combined carbohydrate or fat restriction with calorie restriction preventing clear determination of the effects of the two interventions.

According to the available studies, both LCD and LFD have same effects on liver enzymes in patients with NAFLD. It seems that low caloric diets regardless of their fat and carbohydrate composition are more effective for reduction in liver enzymes. Hypocaloric diets are associated with insulin resistance and metabolic syndrome improvement, so they are effective in reversal of the changes seen with NAFLD. Both LCD and LFD were able to reduce serum transaminase levels but greater improvement appears to be seen with LFD (30).

###### Effects of LCD or LFD on liver fat content

Haufe et al. (23) in their comparative study showed that the reduction of liver fat following hypocaloric LCD (CHO: 25%, FAT 45%) and hypocaloric LFD (FAT: 27%, CHO: 52%) was similar. Patients with higher baseline intrahepatic fat (IHF) achieved a greater reduction of IHF in this study. Kirk et al. (14) showed that after 48 h, IHF decreased more with the LCD (CHO: 10%, FAT: 75%) than LFD (FAT: 20%, CHO: 65%) but after ~11 weeks the reduction was similar in both groups.

Mardinoglu et al. (25) performed an isocaloric very LCD (CHO <10) diet for 10 obese patients with NAFLD. From the first day of intervention, liver fat content was dramatically educed and the mean reduction was 43.8% after 2 weeks. Yu et al. (33) conducted a single arm study (CHO <10%, 800 kcal) and demonstrated a liver fat content reduction of two thirds after 8 weeks (*p* = 0.004).

Marina et al. (31) in a comparative study showed LFD (fat = 20%) could reduce liver triglyceride content but it was not significant in comparison to a high fat diet (LCD).

Browning et al. (21) concluded that after 2 weeks liver fat content decreased significantly with weight loss (*P* < 0.001) but decreased significantly more in LCD (*P* = 0.008). In a study involving XX subjects, managed with a 4 week intervention with very LCD (CHO = 14%), Benjaminov et al. (32) also showed liver fat content reduction.

In comparing LFD and MD, Ryan et al. (36), in a 6-week crossover design incorporating LFD (FAT: 30%, CHO: 50%) or MD (CHO: 40%, FAT: 35–40%), showed a significantly greater reduction in IHF with MD than LFD (*p* = 0.03). However, in a parallel design Properzi et al. (35) observed no difference in liver fat content between groups (*p* = 0.32) with mean (SD) relative reductions in LFD and MD being 25.0 and 32.4%, respectively. Regardless of energy intake, it seems both LFD and LCD can reduce IHF in NAFLD patients.

## Discussion

### Principal findings

This systematic review evaluating low fat vs. low carbohydrate diets for alcoholic fatty liver disease has demonstrated several major findings. Firstly, considering that NAFLD is one of the commonest causes of liver disease worldwide, there is an overall lack of consensus on which dietary intervention is most beneficial for these patients. Secondly, there is also an overall lack of consensus on the definition of the different restrictive diets and the percentage of macronutrient restriction recommended. Moreover, although most of the included papers relate to trials in middle- or high-income counties the diagnostic methods for NAFLD varied. With these caveats in mind, it appears that both LCD and LFD have the same effects on liver transaminases in individuals with NAFLD and that low calorie diets, regardless of their fat and carbohydrate composition, are more likely to read to reduced transaminases.

Hypocaloric diets are associated with improvements in insulin resistance and metabolic syndrome, and consequently have beneficial effects in NAFLD. Both LCD and LFD are able to reduce serum transaminase levels, however, this improvement tends to be more marked in LFD (30). Additionally, both LFD and LCD seemed to have report weight loss results in patient groups. Moreover, two studies reported fever impact of low free sugar diet on NAFLD in adolescent boys (41) and adults (42). Lastly, there remains insufficient evidence to deduce what duration, combination or intensity of treatment is most effective in these patients.

### Limitations

There are several limitations present in this review. Firstly, the included studies are not homogenous. The authors defined low carbohydrate, low fat and low-calorie diets using different parameters. In the included studies, the percentage restrictions ranged from 8 to 45% for LCD and 15 to 30% for LFD. This can affect the reliability of this systematic review. Moreover, it may be difficult to distinguish results between a low calorie, low carbohydrate and low-fat diet, as most of these diets restricted calories and both macronutrients. Another limitation of this systematic review is that most studies did not explore or categorize patients according to different characteristics. For example, patients with different severities of NAFLD may have responded differently to different types of diet adjustments. Similarly, patients with higher body mass indexes may have more marked reductions in weight or liver fat content than their thinner counterparts. This is an increasingly important limitation now that medicine continues to become more individualized. Lastly, the follow up time period varied amongst included studies, with the shortest being 2 weeks and the longest 24 weeks. Therefore, the long-term clinical outcomes of these diets including weight loss, liver enzymes, liver fat content and patient compliance, remain unexplored.

### Comparison with previous literature

The current results agree with previous reviews in that calorie restricted diets are overall successful in reducing, but rarely resolving, NAFLD (43, 44). One previous trial compared hypocaloric LFD and LCD for intrahepatic fat reduction in NAFLD and found no significant difference between the beneficial effects of these two diets over a period of 6 months (23). The current results reflect these outcomes and reiterate the noteworthy impact that dietary modification can have on these markers of liver disease.

Additional large studies have also demonstrated that multidisciplinary lifestyle modification, including exercise and diet, can have an even more pronounced impact on NAFLD with one cohort achieving a 64% remission rate after 12 months (45, 46). Nevertheless, other reviews mention the difficulty of managing NAFLD patients with dietary interventions outside of highly controlled trial settings. These interventions require high-intensity specialist services that may not be available, and, whose cost-effectiveness may need to be explored (43).

This systematic review has contributed to existing literature by summarizing the evidence on LCD vs. LFD and confirming that both of these are demonstrated to be successful interventions to promote weight loss and improve hepatic markers in NAFLD.

### Suggestions for further research

Future research is needed to answer the questions elicited by the limitations of this report. For example, the intensity of macronutrient restriction that generates the most beneficial response in patients. Secondly, the duration of treatment needed, and therefore, both the feasibility and effectiveness of long-term dietary interventions for the management of NAFLD. Thirdly, the current review suggests that LFD may be more successful in reducing transaminases in these patients, therefore, further exploration of this effect is warranted. Another important component of lifestyle intervention targeting NAFLD is physical exercise. Future research will need to further explore the ideal combination between physical exercise and dietary interventions to ensure long term positive outcomes for these patients.

## Conclusions

All calorie restrictive dietary interventions are beneficial for reducing weight, liver fat content and liver enzymes in individuals with NAFLD. Low fat diets seem to be markedly successful in reducing transaminase levels. Further research is needed to explore diet intensity, duration and long-term outcome.

## Data availability statement

The original contributions presented in the study are included in the article/Supplementary material, further inquiries can be directed to the corresponding author/s.

## Author contributions

EK, KA, HV, and AH designed the study. MM, MA, FA, AA, and YS contributed to the literature search, screening data, and data extraction. AA-Z and HK carried out the quality assessment. GR and AS analyzed and interpreted data. HV, FP, AS, AH, and AA-Z wrote and edited the manuscript. All authors read and approved the final manuscript.

## Funding

This study was supported by grants from the Student Research Committee, Shahid Beheshti University of Medical Sciences (SBMU), Tehran, Iran (Grant's ID: 1398/9924).

## Conflict of interest

The authors declare that the research was conducted in the absence of any commercial or financial relationships that could be construed as a potential conflict of interest.

## Publisher's note

All claims expressed in this article are solely those of the authors and do not necessarily represent those of their affiliated organizations, or those of the publisher, the editors and the reviewers. Any product that may be evaluated in this article, or claim that may be made by its manufacturer, is not guaranteed or endorsed by the publisher.
