# Supplementary material for: Low fat diet versus low carbohydrate diet for management of non-alcohol fatty liver disease: A systematic review
Source: Front Nutr. 2022 Aug 16;9:987921. doi: 10.3389/fnut.2022.987921 (PMC9424761; doi:10.3389/fnut.2022.987921)
Supplement: Supplementary file 1 [file Table_1.docx]

| **Supplementary Table 1.** Search strategy. |
| --- |
| **Pubmed: 266**  ((((((((((("Diet, Fat-Restricted"[MeSH]) OR "Fat-Restricted Diet*"[Title/Abstract]) OR "low-fat"[Title/Abstract]) OR "fat-free diet*"[Title/Abstract]) OR "Diet, Carbohydrate-Restricted"[MeSH]) OR "Carbohydrate-Restricted Diet*"[Title/Abstract]) OR “low-carb*”[Title/Abstract]) OR "low-fructose"[Title/Abstract]) OR "low-sucrose"[Title/Abstract]) OR “ketogenic diet”[Title/Abstract])) AND (((((((((((((((((("Liver"[MeSH ]) OR "Liver"[Title/Abstract]) OR “Non-alcoholic Fatty Liver Disease”[MeSH ]) OR “Non-alcoholic Fatty Liver Disease”[Title/Abstract]) OR “Liver Diseases”[MeSH ]) OR “Liver Diseases”[Title/Abstract]) OR “End Stage Liver Disease"[MeSH ]) OR “End Stage Liver Disease"[Title/Abstract]) OR “fatty liver*”[MeSH ]) OR “fatty liver*”[Title/Abstract]) OR “NAFLD”[Title/Abstract]) OR “NASH”[Title/Abstract]) OR “Nonalcoholic steatohepatitis”[Title/Abstract]) OR “nonalcoholic fatty liver*"[Title/Abstract]) OR “nonalcoholic steatohepatitis*”[Title/Abstract]) OR “alcoholic fatty liver”[Title/Abstract]) OR “Fatty Liver, Alcoholic”[MeSH ]) OR “steatosis*”[Title/Abstract])) AND ((((((((((((((((“Clinical Trials as Topic”[MeSH ]) OR “Cross-Over Studies”[MeSH ]) OR “Double-Blind Method”[MeSH ]) OR “Single-Blind Method”[MeSH ]) OR “Random Allocation”[MeSH ]) OR RCT[Title/Abstract]) OR “Intervention Studies”[Title/Abstract]) OR “intervention”[Title/Abstract]) OR “controlled trial”[Title/Abstract]) OR “randomized”[Title/Abstract]) OR “randomised”[Title/Abstract]) OR “random*”[Title/Abstract]) OR “randomly”[Title/Abstract]) OR “placebo”[Title/Abstract]) OR “assignment”[Title/Abstract]) OR "clinical trial"[Title/Abstract]) |
| **Scopus: 718**  ( ( ( TITLE-ABS-KEY ( "Fat-Restricted Diet*" ) OR TITLE-ABS-KEY ( "low-fat" ) OR TITLE-ABS-KEY ( fat-free AND diet* ) OR TITLE-ABS-KEY ( carbohydrate-restricted AND diet* ) OR TITLE-ABS-KEY ( low-carb* ) OR TITLE-ABS-KEY ( "low-fructose" ) OR TITLE-ABS-KEY ( "low-sucrose" ) OR TITLE-ABS-KEY ( "ketogenic diet" ) ) ) AND ( ( TITLE-ABS-KEY ( "Liver" ) OR TITLE-ABS-KEY ( "Non-alcoholic Fatty Liver Disease" ) OR TITLE-ABS-KEY ( "Liver Diseases" ) OR TITLE-ABS-KEY ( "End Stage Liver Disease" ) OR TITLE-ABS-KEY ( "fatty liver*" ) OR TITLE-ABS-KEY ( "NAFLD" ) OR TITLE-ABS-KEY ( "NASH" ) OR TITLE-ABS-KEY ( "Nonalcoholic steatohepatitis" ) OR TITLE-ABS-KEY ( "nonalcoholic fatty liver*" ) OR TITLE-ABS-KEY ( "nonalcoholic steatohepatitis*" ) OR TITLE-ABS-KEY ( "alcoholic fatty liver" ) OR TITLE-ABS-KEY ( "steatosis*" ) ) ) AND ( ( TITLE-ABS-KEY ( "clinical trial" ) OR TITLE-ABS-KEY ( cross-over ) OR TITLE-ABS-KEY ( double-blind ) OR TITLE-ABS-KEY ( single-blind ) OR TITLE-ABS-KEY ( "Random Allocation" ) OR TITLE-ABS-KEY ( rct ) OR TITLE-ABS-KEY ( intervention ) OR TITLE-ABS-KEY ( "controlled trial" ) OR TITLE-ABS-KEY ( randomized ) OR TITLE-ABS-KEY ( randomised ) OR TITLE-ABS-KEY ( random* ) OR TITLE-ABS-KEY ( randomly ) OR TITLE-ABS-KEY ( placebo ) OR TITLE-ABS-KEY ( assignment ) ) ) |
| Web of science: 343  (TS=("Fat-Restricted Diet*") OR TS=("low-fat") OR TS=(fat-free diet*) OR TS=(Carbohydrate-Restricted Diet*) OR TS=(low-carb*) OR TS=("low-fructose") OR TS=("low-sucrose") OR TS=("ketogenic diet")) AND (TS=("Liver") OR TS=(“Non-alcoholic Fatty Liver Disease”) OR TS=(“Liver Diseases”) OR TS=(“End Stage Liver Disease") OR TS=(“fatty liver*”) OR TS=(“NAFLD”) OR TS=(“NASH”) OR TS=(“Nonalcoholic steatohepatitis”) OR TS=(“nonalcoholic fatty liver*") OR TS=(“nonalcoholic steatohepatitis*”) OR TS=(“alcoholic fatty liver”) OR TS=(“steatosis*”)) AND (TS=("clinical trial") OR TS=(Cross-over) OR TS=(Double-Blind) OR TS=(Single-Blind) OR TS=("Random Allocation") OR TS=(RCT) OR TS=(intervention) OR TS=("controlled trial") OR TS=(randomized) OR TS=(randomised) OR TS=(random*) OR TS=(randomly) OR TS=(placebo) OR TS=(assignment)) |
| **Cochrane: 130**  (("Diet, Fat-Restricted"):ti,ab,kw OR ("low-fat"):ti,ab,kw OR ("Fat-Restricted diet"):ti,ab,kw OR ("fat-free diet*"):ti,ab,kw OR ("Diet, Carbohydrate-Restricted"):ti,ab,kw OR ("low-carb*"):ti,ab,kw OR ("Carbohydrate-Restricted diet*"):ti,ab,kw OR ("low-fructose"):ti,ab,kw OR ("low-sucrose"):ti,ab,kw OR ("ketogenic diet"):ti,ab,kw) AND (("liver"):ti,ab,kw OR ("non-alcoholic fatty liver disease"):ti,ab,kw OR ("liver disease"):ti,ab,kw OR ("end stage liver disease"):ti,ab,kw OR ("fatty liver"):ti,ab,kw OR ("alcoholic fatty liver"):ti,ab,kw OR ("NAFLD"):ti,ab,kw OR ("NASH"):ti,ab,kw OR ("nonalcoholic steatohepatitis"):ti,ab,kw OR ("nonalcoholic fatty-liver disease"):ti,ab,kw OR ("steatosis hepatis"):ti,ab,kw OR ("steatosis*"):ti,ab,kw) |
